# From monitoring to action: utilising health survey data in national policy development and implementation in Finland

**DOI:** 10.1186/s13690-019-0374-9

**Published:** 2019-11-13

**Authors:** Katri Kilpeläinen, Päivikki Koponen, Hanna Tolonen, Seppo Koskinen, Katja Borodulin, Mika Gissler

**Affiliations:** 1 Department of Welfare, Finnish Institute for Health and Welfare, Welfare and Health Promotion Unit, Mannerheimintie 166, FI- 00270 Helsinki, Finland; 2 Department of Public Health Solutions, Finnish Institute for Health and Welfare, Mannerheimintie 166, FI- 00270 Helsinki, Finland; 3 Age Institute, Jämsänkatu 2, FI- 00520 Helsinki, Finland; 4 Department of Information and Service, Finnish Institute for Health and Welfare, Mannerheimintie 166, FI- 00270 Helsinki, Finland; 5 Department of Neurobiology, Care Sciences and Society, Karolinska Institute, SE- 171 77 Stockholm, Sweden

**Keywords:** Health surveys, Public health surveillance, Health promotion, Health policy, Finland

## Abstract

**Background:**

Health interview and examination surveys provide valuable information for policy, practice and research purposes. Appropriate use of high-quality, representative and timely population data can indirectly help the citizens to live healthier and longer lives. The aim of this study was to review how health survey data have supported health policy making, health research and everyday health care in Finland.

**Methods:**

Data were collected by focused interviews with ten Finnish senior experts from the Ministry of Social Affairs and Health, political parties, National Institute for Health and Welfare, universities, and health associations.

**Results:**

Most interviewees agreed that health surveys have positively affected the health of the population over the past 50 years - through health strategies, care guidelines, legislation, research, prevention programs, risk calculators, and healthier products on the market. There is also a need for further development: the latest research results should be provided in a nutshell for politicians, and effective tools should be developed more for health care professionals’ use. The coverage of health information on children, adolescents, oldest old, disabled persons, migrants and ethnic minorities should be improved.

**Conclusions:**

Sound health policy and its successful implementation require extensive national cooperation and new communication strategies between policy makers, researchers, health care professionals, health service providers - and citizens. The future health information system in Finland should better cover all population groups. To obtain more comprehensive health information, the possibilities for register linkages should be secured and register data should be further evaluated and developed to serve health monitoring purposes.

## Background

Finland is globally one of the forerunners in the field of monitoring adults’ health. Finland has a tradition of 50 years to gather information about the health and welfare of Finnish adults on the population level. Surveys and administrative registers form a basis for the national health monitoring. Health monitoring system has been built to support targeting resources toward identified problem areas, and to predict future needs for health care and preventive measures [1–8]. The North Karelia Project is a prime example of prevention programmes that have gained great improvements in population’s health [9, 10].

Information on mortality, health status and health care is obtained from administrative health registers covering the whole country and all ages. Health care registers only cover those individuals who have used services, and they include very limited information on the individual’s background. Health surveys complement the register information. Health interview surveys (HIS) provide self-reported information on perceived health, lifestyle, socio-economic situation, opinions, health service needs and service utilisation. Key information on risk factors and functional capacity can only be collected by Health examination surveys (HES), which include questionnaire-based data, physical measurements and biological sample collection [1, 5–8, 11–14].

Both HIS and HES have been carried out at regular intervals in Finland for several decades. The HES tradition was started by mobile clinic health examination surveys in different parts of the country in 1966 to 1976. The National FINRISK Study (HIS/HES) was carried out every fifth year between 1972 and 2012, and three more comprehensive national surveys between 1978 and 2011 (the Mini-Finland Survey, the Health 2000 and the Health 2011 Surveys). These have recently been merged into the FinHealth Study since 2017. The national HIS tradition was started by the Social Insurance Institution, which initiated a series of interview surveys on social and health security in 1964, 1968 and 1976. The national HIS on health behaviour was carried out annually between 1978 and 2014. The HIS were later developed to cover a wider focus and larger samples (the Regional Health and Well-being Study between 2010 and 2017, and the national FinSote survey of health, well-being and service use since 2017). The School Health Promotion (SHP) study has provided information on the 14—20-year-old Finnish adolescent’s wellbeing since 1996. Since 2010, a few surveys have been focused to migrants and persons with foreign origin [1, 5–8, 11–14].

Our study aims to review how health survey data have supported Finnish health policy, how these data could be better utilised, and how to guarantee the comprehensive health information system also in the future. The use of health survey data has rarely been evaluated, but a few analyses from England have been reported [15–17].

## Methods

Data were gathered by ten interviews with senior experts (seven women and three men). They were selected to represent different experiences in collecting, analysing and/or reporting major national health surveys and/or utilizing health survey data and developing health policy. They represented the Ministry of Social Affairs and Health (two experts utilizing health survey results), political parties (one expert with a long experience in both research and policy development), the National Institute for Health and Welfare (four research/survey experts with also experience in developing and evaluating national and international health services, national programmes and policy), one university professor (survey expert), and two Finnish Non-Governmental Patient Associations (experts for major public health problems). The interviews were carried out between July 2016 and February 2017 and they were held in Finnish by the main author. Two other investigators (HT and PK) participated to three interviews. None of the invited experts refused. Five experts were interviewed face-to face, one by video contact, and one by telephone. Three experts preferred to reply by e-mail.

Focused interview technique was used to collect qualitative data [18]. The interviews were based on three main themes: 1) What are the most important advantages of national health surveys; 2) How could health survey data be more extensively utilised; 3) How to guarantee the comprehensive health information system also in the future. Targeted questions about the predetermined categories were followed by the open-ended questions. Also further questions aroused during the interview (“You said a moment ago … can you tell me more?”), and those were asked also from the e-mail respondents. The interviews lasted from 30 to 90 min. The average length of the written e-mail responses was one page. All documents mentioned by the experts were reviewed.

**Fig. 1:**
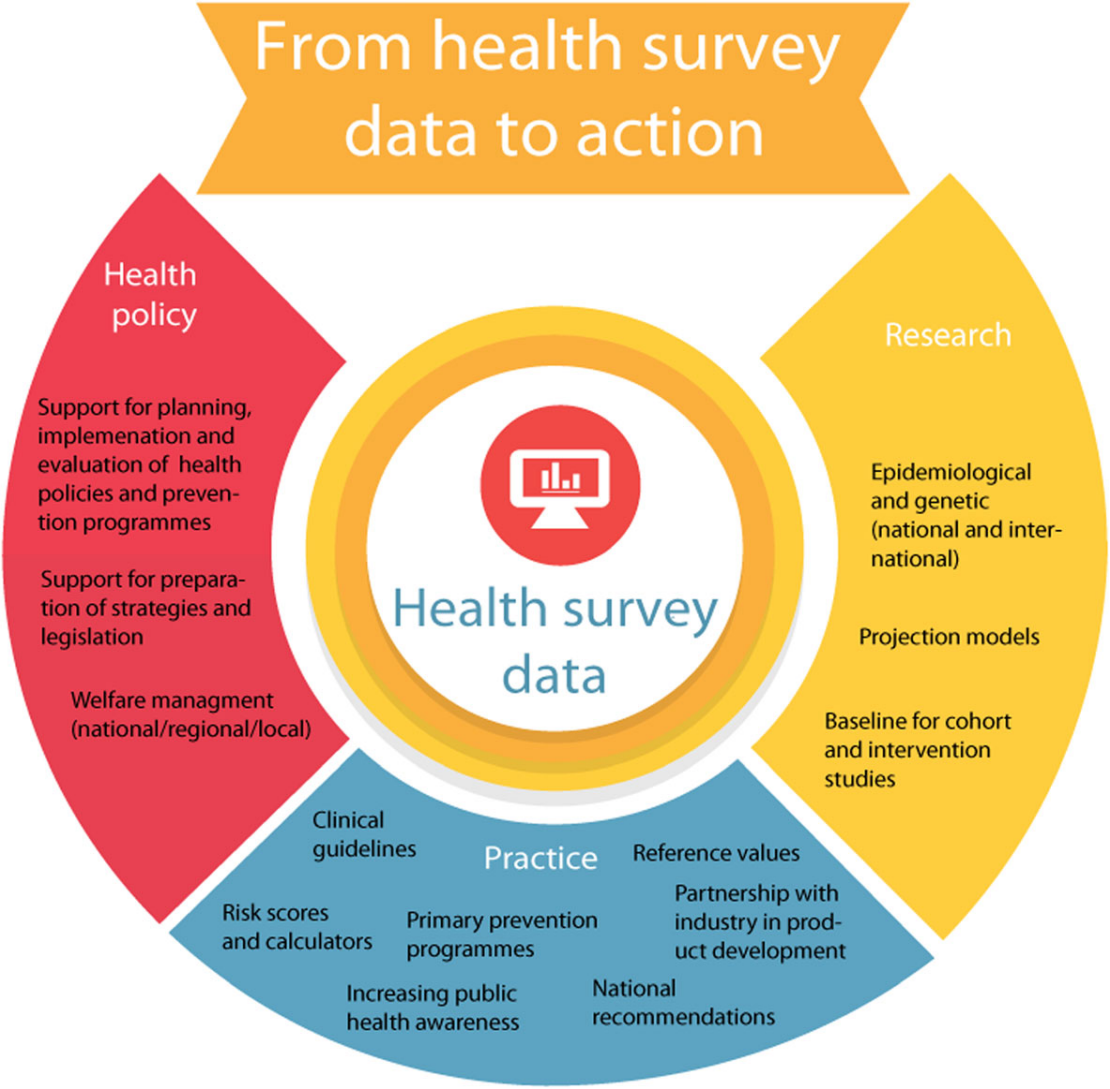
Health survey data utilisation in health promotion through health policy, health care practices and research

Deductive content analysis was used to analyse the data [19]: 1) The audio material was partly transcribed during and after the interviews. The first author listened and read through the audio and text materials multiple times to obtain a sense of the whole data. In total 68 codes were identified from the data and these were further classified into 13 subcategories (Additional file 1); 2) All members of the study team checked the categories and further developed the subcategories in shared discussions, and finally decided to classify them into the three key categories: policy, practice and research (Fig. 1); 3) The need of merging or further sub-categorising was checked; 4) The original transcripts were read and listened once again to ensure that all the information was included; 5) The manuscript was shown to all experts to enable them to comment on whether any important examples had been missed. Their feedback was taken into account in finalising the manuscript.

There was no need to submit the study to the Institutional Ethics Committee of THL, because the interviews were considered as part of the regular core tasks of THL to evaluate the data produced by the institution. The THL Ethics Committee has decided that such interviews conducted as part of regular daily work and where there is no potential harm to the voluntary participants, do not need a formal ethics approval. This is in line with the national guidelines. Verbal informed consent was obtained from all participants instead of written, as all interviewed experts were considered as collaborators in this regular evaluation, not primarily as research subjects, and they all checked the final manuscript. They were asked to check whether there were any incorrect interpretations or if any important examples had been missed, and their comments have been taken into account when finalizing the manuscript. Thus, they all agreed that the results can be published.

## Results

Overall, respondents consistently felt that the Finnish health surveys provide valuable data for health policy, health care practice and research (Fig. 1, Additional file 1).

### Policy

Policy was a general theme in all interviews. All experts agreed that analyses based on health survey data have been widely utilised in developing, implementing and monitoring the Finnish health policy. In general, evidence-based health policy was supported.

“*Health survey information help to target resources toward identified problem areas. Without the evidence-based information health policy would be just “guessing policy*”.

Besides getting information to support decision-making, the importance to evaluate health effects after these decisions had been made was identified.

*“Survey information is needed also when evaluating the Acts, strategies and reforms afterwards - to look back what good we have reached*”.

The experts pointed out that legislation is among the most powerful policy instruments. They perceived that health surveys have influenced the taxation policy in Finland.

“*Health surveys have shown that prices of alcohol and tobacco have a strong effect on their consumption. These findings have been taken into account in decisions concerning the taxation of these produc*t*s*.”

The importance of local level survey information in welfare management was also raised.

“*Reliable, comparable and up-to-date health data are needed at local level. We need it to compare our own situation to other municipalities and regions, preparing the obligatory municipality and regional welfare reports, and implementing the local welfare management and health promotion in general.”*

### Practice

Practice was another general theme in the interviews. National health promotion programmes and recommendations are based on the health survey data, and they were mentioned by all experts as the main tools to implement health strategy.

*“Prevention programs and recommendations for different purposes have steered concrete health promotion actions in national, regional and local level in Finland. Examples of the topics are nutrition, leisure time physical activity, musculoskeletal disorders, obesity, allergy, asthma, alcohol, oral health, and cognitive capacity.”*

Clinical Care Guidelines (currently available for 108 health problems and medical treatments) were mentioned by all experts. They agreed that health survey data have provided epidemiological background information for those guidelines.

*“Care guidelines are the basis for treatment decisions. They are concrete tools for physicians, healthcare professionals and citizens every day.”*

Collaboration with food industry was mentioned in several interviews.

*“As vitamin D intake fell below recommendations according to the 2002 study, a systematic vitamin D fortification of fluid milk products and fat spreads was started in 2003 with good results. Moreover, too low levels of iodine were successfully stabilised by supplementing table salt and bread products with iodine.”*

*“Food industry follows eagerly the Finnish Nutrition Recommendations, and this has led to innovative health product developments. Examples are the Heart Symbol products that are healthier choices in their product group, lactose free dairy products, and plant stanol ester yogurts and margarines which have been proven to lower cholesterol levels.*”

Many risk calculators have been developed based on health survey data. The experts perceived that health professionals have been able to use the calculators to identify risks and monitor the treatment of high-risk individuals, and citizens have been able to estimate their personal risks (especially the new web-based versions and mobile applications). There was a consensus on their importance.

“*FINRISK calculator is one of the most significant risk score tools. Other examples of developed risk calculators are FINDRISK for type 2 Diabetes and risk calculator for dementia. All these can reveal persons in high-risk, and these individuals can be advised to contact a doctor for further treatments. They can encourage users to actively decrease their modifiable risk factors. There are, for example, almost 150 000 Finns who are not aware of their type 2 diabetes. Many lives could be saved if those persons were found and treated”.*

The experts perceived that Finland has been one of the global forerunners in the field of health surveys, as Finnish experts have led many multi-country health monitoring projects.

*“The Finnish model, where different data sources are widely used for health monitoring purposes, has provided many concrete tools for improving health information systems around the world.*”

Media has given much attention to health surveys, and its strong influence on citizens, politicians and private sector was mentioned by many experts.

“*Opinions and behaviour changes of the population have a strong influence on politicians and private sector through media, because*” *voters decide” and*” *consumer is a king”. The power of media is much stronger than any public health promotion campaign.”*

### Research

Research was a general theme in interviews with survey experts. They highlighted that health survey data are a basis for epidemiological research, which is essential in evidence-informed welfare management and policy.

“*The best possible information for policy development should always be guaranteed. For example reducing health inequalities is a complex issue, which actions need strong knowledge base and thus, strong knowledge base is needed for action.”*

The experts perceived that in addition to providing data for epidemiological and genetic research, health surveys have provided foundations for many topic specific sub-studies, cohort studies and intervention studies. In such studies, the original survey data has given baseline information or the general population reference group for certain patient groups. Some experts admitted that it may be difficult to fulfil all health information needs.

“*As health surveys are expensive to carry out, they do not cover all fields of human health. Therefore, additional surveys with selected baseline data from the previous population-based health survey participants, are welcome. On the other hand, the researchers are thankful to get a general population reference data for their own patient data. A win-win situation.”*

Many experts pointed out that survey data collected during several time periods to show trends, has led to developing projections.

*“Projection models can assess for example how functional capacity of the elderly, or the prevalence of diabetes will develop in the future if adequate preventive actions take place. There is a huge potential to save money. At least 80% of coronary heart disease, over 90% of diabetes, 30% of cancer, and a large part of respiratory diseases could be prevented. Dementia could be moved 5-10 years forward by active lifestyle counselling.”*

DNA has been collected from the HES participants since 1992, and the experts perceived that this has been beneficial for the Finns in many ways.

*“Genetic analyses of the FINRISK study have contributed to identification of genetic variants associated with common public health problems, and also with rare hereditary diseases. This information can help doctors to diagnose and treat patients. Examples of the latter are familial hypercholesterolemia and other familial dyslipidemias, long QT syndrome (LQTS), and increased risk for colon or breast cancer, which might lead to death at early ages. By identifying these patients, prevention can be enhanced and lives can be saved*.”

The experts acknowledged that research groups around the world have used the Finnish health survey data for their studies*.*

*“The high quality Finnish health data are well known, and therefore they are a part of many multi-country projects.*”

### Future challenges

The interviews revealed several challenges in monitoring health in Finland. The importance of developing health monitoring for the oldest old, disabled persons, migrants and ethnic minorities were mentioned by two experts. Most experts highlighted the importance of developing health monitoring among children and adolescents.

“*It is essential to ensure that children are healthy and have the opportunities to fulfil their potential. Prevention is not possible if there is no information what should be prevented.*”

All respondents felt that evidence-based information should be made more visible for politicians and other stakeholders.

“*Political stakeholders would appreciate more concrete results, guidelines and tools on how to implement the health programs in practise*. *Politicians are not specialists in all areas, and they do not have time to get acquainted with all issues.”*

*“As at local level there are small resources for health promotion, all concrete guidelines are welcome. Tools such as the National Obesity Programme should be available for different topics.”*

The experts perceived that media has raised challenges to health promotion.

“*Experts participate in media discussions more actively than previously, and most people trust in experts’ opinions, but there is an increased amount of alternative views and critical debate in health promotion.* S*ometimes the focus on health is less predominant, and the messages do not go in line with official recommendations. The low carbohydrate diets, increased consumption of saturated fat, and critical attitudes against vaccinations are examples of those discussions.”*

The experts reported that laboratory reference values in Finland are currently mostly based on hospital patient data, not on data from the general population. They felt that hospital data have limitations as not all citizens use health services – even though the public health care services are offered to all citizens.

“*The laboratory reference values should definitely be based on the reality – the population with and without the health problems. Health surveys can provide information on both”.*

Although more than one thousand scientific studies have been published based on the Finnish health survey data, most experts felt that they are not utilised to their full potential.

“*National Institute for Health and Welfare provides quality ready-to-use data and support for researchers. This opportunity should be utilised as much as possible in doctoral thesis, post-doctoral studies and product development. For example increased use of genetic information could help to identify the persons who are at high risk of developing the non-communicable disease. These persons could then receive lifestyle counselling, and also tailored medication if needed.”*

Expensiveness of the health surveys was highlighted by two experts. Wider use of register-based information and Big data, and wider collaboration with the private sector were suggested to save costs.

“*Instead of expensive health surveys, why are health data not collected as part of the services, and why “Big data” are not used to understand and predict the behaviour of the population like private companies do? For example, grocery shopping data, Google searches, or information received from the fitness trackers could potentially give valuable information also on those who do not use health services, or do not participate in surveys.”*

“*Health surveys are needed but they should not include all population groups every time, and private companies should be activated to fund parts of the health surveys in exchange for obtaining data for their specific needs.”*

## Discussion

### Main results

Most experts agreed that health surveys have affected positively the health of the population through health strategies, national recommendations and health promotion programmes, clinical guidelines, laws, research, risk calculators, and healthier products at the markets. All are used, alone and in varying combinations, in developing and evaluating public health policy. The main challenges are under-use of health survey information by political decision-makers and health care professionals, and poor coverage of information on children, adolescents, the oldest old, and specific population groups such as disabled persons, migrants and ethnic minorities.

### Strengths and weaknesses

Due to a long history of health surveys in Finland, it was easy to identify several experts for the focused interviews and to obtain detailed responses. The experts were selected based on their work with health surveys and health policy. The subjective views of the respondents, most with a long experience on health surveys, are potential weaknesses of the study. The respondents might have undermined critical views and the benefits of other data sources in health monitoring (e.g. administrative and register data). The critical views for example on preferring other data sources, should be explored further in an additional selection of persons with different types of expertise in public health and health policy.

Credibility of the study was supported by several ways: by asking several questions regarding each topic, by encouraging the participants to support their statements with examples, by asking follow-up questions from all participants, by asking all interviewed experts to check the final manuscript whether there were incorrect interpretations or if any important examples had been missed, and by editing the final manuscript based on their feedback. The number of interviewed persons was small, but saturation in the responses was observed as the same aspects were reported by more than one expert. Analyst triangulation was applied by using observer in three interviews, and by a dialogue in reviewing and developing the categories. In the team, different researchers pointed out several aspects before a consensus on the final categorization was reached.

The analysis was supported by critical questions and comments from those co-authors who were unware of the identity of the experts. This promotes dependability of the study. Data triangulation was applied by using the various data sets throughout the analysis process: mainly interview data and documents raised up by the interviewees (scientific articles, project reports, reports on public health programs as well as policy documents) as well as theoretical literature. Transferability was promoted by describing experience of the interviewees so that the results would become meaningful to an outsider. This study focuses on the experiences from selected experts in Finland at the time of the interviews and the transferability of the result to other contexts is limited. Confirmability was supported by describing transparently the study process and reporting the findings with quotes from the original interviews. However, social desirability response bias is possible: the experts might have answered in a manner that they think the interviewer desires. The interviewer (first author) has long experience in health monitoring and health surveys, which could be both a benefit and a source of bias.

### Further needs

The future health information system in Finland should provide reliable information not only on working-aged adults but also on the population groups poorly covered so far. More effective use of data collected at child and school health care, military health care and primary health care should be enhanced. The unique opportunity to link several registers with others, and with survey data, should be secured and further developed. Health surveys are the only source of information on those who do not use health services, and on topics not covered in primary health care records or registers (like needs and access to health care services, functional capacity, and unidentified risks and undiagnosed diseases). When developing laboratory reference values, health survey data should be preferred instead of patient data.

Health survey information should be more promoted to political stakeholders, researchers and health care professionals. Critical reviews and discussions of both the benefits and limitations of health survey data compared to other data sources are also needed. A multi-sectoral advisory board on public health could be a solution for more effective dissemination of survey results, for developing concrete tools to implement public health policy, and for targeting resources to the areas or population groups with the most prevalent health problems. If such a board is aware of the complex realities of policymaking, and focuses on how and to what extent research evidence gets transferred to decision makers, the gap between what the government does, and what the research evidence says they should do, could be narrowed [20, 21].

Political decisions are not entirely based on rational, evidence-based considerations. There are limits to what extent evidence can influence political decisions. The political science perspective on power relationships can help to better understand this policy problem [22–24]. In the context of the Public Health Action Cycle [25], surveys can help in identifying problems, supporting evidence informed decisions for policies to address the problems, and after implementation, in policy evaluation. A dialogue between survey experts with comprehensive public health knowledge and the politicians making decisions on concrete health policy projects and processes is needed.

Finland had a national board in 2003–2015 to coordinate the preparation, implementation and evaluation of the national Health 2015 Programme. It comprised of representatives of a wide spectrum of health promotion organisations, ministries, universities and other authorities and enabled active multi-sectoral collaboration. Needs for a similar new body have been discussed.

Besides finding new ways to reach the stakeholders’ attention, the board could develop communication strategies and new methods to reach those who have critical views on the official health recommendations. Critical debate and a rapid rise of social media are not just challenges, but they also give new ideas and possibilities for untraditional health promotion work.

In many areas of public health a deeper understanding of the key phenomenon is needed, and therefore the researchers and research groups should be encouraged to better utilise health survey data. Individual level health data cannot be openly available for everyone due to the sensitive nature of data, as regulated by law [26]. However, research groups can access the national health survey data in Finland for example through the THL biobank and in the future also via secured remote access systems. Open data requirements can be addressed through interactive, fully anonymized data portals. Survey results have been published in a few such data portals in Finland, but there is an urgent need to develop these further for example like Belgium has done [27]. Traditional reports on survey results as paper or web publications are not sufficient anymore.

Declining survey response rates are a growing international trend and they might increase the risk of nonresponse error. The reasons for this decline are multiple: the rising number and types of surveys, problems in contacting people (e.g. due to mobile phones with private numbers), societal changes, lower trust in public health institutions in certain population groups and greater awareness and concern of privacy issues. Therefore stronger fieldwork efforts different contact attempts and responsive designs are needed in the future [28–30].

During the time of interviews in this study, the data protection legislation was not under active debate in Finland. The 2019 Act on the Secondary Use of Health and Social Data will enhance use of health and social data (e.g. data from electronic patient records) in developing health care services, education, management and supervision in Finland. There have been worries that strict interpretation of the European General Data Protection Legislation (GDPR) may diminish the possibilities for collection Big data sets for research [26]. Since GDPR does not concerns anonymised data, this type of data can be used, as previously, for statistical and research purposes. In general, GDPR promotes the use of data for scientific research also in case when data cannot be made anonymous. There is a special emphasis that data can be used for purposes in public interest, scientific or historical research purposes and statistical purposes.

The use of Big data generated for example from social media behaviour (Twitter, Facebook, Instagram), grocery store purchases, Helpful Numbers, GPS trackers and Google searches – may in the future complement other data sources on individuals’ behaviour. However, at least in the near future they will not replace health surveys due to technical and ethical problems, poor coverage of the main topics in health surveys and the lack of representativeness. Wider use of big data could also include register information, but first the registers need to be developed to enhance representativeness and validity of the information they cover [31].

## Conclusions

Sound health policy and its successful implementation require extensive national cooperation and new communication strategies between policy makers, researchers, health care professionals, health service providers - and citizens. The future health information system in Finland should better cover all population groups. To obtain more comprehensive health information, the possibilities for register linkages should be secured and register data should be further evaluated and developed to serve health monitoring purposes. The data protection regulations need to be implemented to secure privacy of individuals, but at the same time enhance wide use of different data sets to support public health.

## Supplementary information

**Additional file 1.** Utilisation of Finnish health survey data in national health policy development and implementation: documents, tools and studies mentioned by the experts.

## Data Availability

The datasets (interviews) used and analysed during the current study are available from the corresponding author on reasonable request.

## Abbreviations

DNA: Deoxyribo Nucleic Acid. The chemical present at the centre of the cells of living things, that controls the structure and purpose of each cell and carries genetic information during reproduction
GPS: Global Positioning System. A system that can show the exact position of a person or thing by using signals from satellites
HIS: Health interview survey
HES: Health examination survey
LQTS: Long QT syndrome. A condition which affects repolarization of the heart after a heartbeat. This results in an increased risk of an irregular heartbeat which can result in palpitations, fainting, drowning, or sudden death
THL: National Institute for Health and Welfare to Finnish Institute for Health and Welfare
WHO: World Health Organisation
