# Supplementary material for: From monitoring to action: utilising health survey data in national policy development and implementation in Finland
Source: Arch Public Health. 2019 Nov 13;77:48. doi: 10.1186/s13690-019-0374-9 (PMC6852713; doi:10.1186/s13690-019-0374-9)
Supplement: Supplementary file 1 — Additional file 1. Utilisation of Finnish health survey data in national health policy development and implementation: documents, tools and studies mentioned by the experts. [file 13690_2019_374_MOESM1_ESM.pdf]

APPENDIX 1. Utilisation of Finnish health survey data in national health policy development and implementation: documents, tools and studies mentioned by the experts.

POLICY

National health strategies and legislation

- National health strategies and legislation (e.g. the most recent Finnish Health strategy, Health 2015) (1,2)
- Health Acts (e.g. Tobacco Act, Alcohol Act, Health Care Act, Primary Health Care Act, Local Government Act, Act on care services for the elderly) (3-9)
- Reports on the status of the Health in Finland at the request of the Ministry of Social Affairs and Health (10-16)
- Local welfare monitoring (e.g. obligatory welfare reports, welfare management, implementation plans, comparisons to other municipalities and regions) (5,6)
- Influence on the tax policies (e.g. sugar, alcohol, tobacco) (17)
- Evaluation of the programmes and Acts (e.g. Health 2015 Public Health Programme (2), the Act for Elderly Care and Services in Finland (18), Health and social services reform in Finland, of which implementation starts in 2021 (19)

International collaboration

- WHO 2020 strategy - a European policy framework and strategy for the 21st century, has been developed on the basis of the Finnish experiences (20)

PRACTICE

Clinical Guidelines

- Clinical Care guidelines for 108 health issues (e.g. diabetes, hypertension, tobacco dependence and cessation, treatment of alcohol abuse, dental caries, asthma, and obesity) (21)

National health promotion programmes and recommendations

- National recommendations and programs for nutrition (22-24), health-enhancing physical activity (18,25-31), musculoskeletal disorders (32), obesity 2012-2018 (33,34), type 2 diabetes (35,36), allergy and asthma 2008-2018 (37), memory and cognitive capacity 2012-2020 (38), sexual and reproductive health (39)
- The Action plan on Alcohol, Tobacco, Drugs and Gambling (40)
- Local and national health promotion projects (e.g. North Karelia Project) (41-44)

Risk calculators validated by health survey data

- FINRISK Calculator. A tool to assess total risk for Coronary Heart Disease and Stroke. (45)
- FINDRISC Diabetes Risk Calculator. A tool to identify high-risk individuals for a Type 2 Diabetes. (46,47)
- Risk score for assessing Dementia risk. A tool to assess risk factors 20 to 30 years before the dementia diagnosis. Available also as mobile application. (48)

Media

- Media use health survey data widely, and it´s power is much stronger than any public campaign. (49)

Partnerships with industry in product development

- Developing products according to the National recommendations and programs for nutrition (50)
- Valio Eila© -grocery brand, lactose free dairy products (51)
- Benecol© -grocery brand (52,53)
- Heart Symbol –better choice© – grocery brand (54,55)

International collaboration

- Finnish prevention programs have led the way to many international initiatives. E.g. a) Experiences of the North Karelia Project have contributed the WHO and other preventing strategies of CVDs and non-communicable diseases around the world (56), b) Finnish Type 2 Diabetes prevention programs have been adapted to many other countries (35, 36), c) Decreasing the maximum salt recommendation from 1.2% to 1.1% in Finland has had impact on the recommendations at EU level.
- Development of the European health monitoring. E.g. ECHIM project (57,58), EHES project (59,60), WHO MONICA project (61), WHO Cindi programme (62), and Finbalt Health monitoring survey (41,63). Protocols in these projects are based on the Finnish experiences.

RESEARCH

Basic epidemiological data

- Databases (e.g. Welfare Compass, Terveytemme.fi, Sotkanet.fi) (64-66)
- Basic reports (e.g. FinHealth (67) and The Regional Health and Well-being Study ATH) (68)

Reference values to the population

- Laboratory reference values in Finland are mostly based on hospital patient data, which has limitations as not all citizens use health services. Values should be based on the reality: the population with and without the health problems. Health surveys can provide information on both.

Baseline for cohort and intervention studies

- Health surveys (e.g. FINRISK Study and Health 2011 Study) have provided foundations for many topic specific sub-studies, cohort studies and intervention studies. In such studies, the original survey data has given baseline information or the general population reference group for certain patient groups. Examples: FINGER study (Finnish Geriatric Intervention Study to Prevent Cognitive Impairment and Disability) (48), DILGOM 2007 Survey and its follow-up 2014 (Dietary, Lifestyle, and Genetic determinants of Obesity and Metabolic syndrome) (69), Fishermen Study (70,71), KARA (Karelian Allergy survey 2003-2012) (72), Migrant Health and Wellbeing Survey (Maamu 2010-2012) (73-75)

Projection models

- Health survey-based time trends enable to predict the future health status of the population. Projection models can assess for example how functional capacity of the elderly (18), or the prevalence of diabetes will develop in the future if adequate preventive actions take place (76).

Genetic research

- FINRISK surveys have collected DNA for 34,000 individuals. DNA collections have enabled researchers for example a) to develop genetic tests for genetic mutations like lactose intolerance (77) and familial hypercholesterolemia (78,79), and b) to identify genetic variants associated with common public health problems and with rare hereditary diseases. (80)

International collaboration

- International collaboration projects. E.g. the FINRISK and Health 2000/2011 data are a part of the international Global burden of diseases Study (GBS), which developed future health scenarios for mortality and disability for different age-sex groups, causes, and regions. (81)
- Many research groups around the world utilise the Finnish HES data in their studies. (82)

Open data access: Fineli database as an example

- Fineli is the National Food Composition Database in Finland. It includes over 4,000 foods and 55 nutrient factors. The content of the Fineli database is freely available as open data that software developers can utilise when planning the new mobile applications to nutrition monitoring (83). For example Miils mobile application helps consumers to plan healthy meals. (84)

REFERENCES (APPENDIX 1)

1. Ministry of Social Affairs and Health. Health 2015 public health programme. Brochures of the Ministry of Social Affairs and Health 2001:8eng. Helsinki: Ministry of Social Affairs and Health, 2002.

2. Rotko T, Kauppinen T. Terveys 2015 -kansanterveysohjelman loppuarviointi. Discussion Paper 8/2016. Helsinki: National Institute for Health and Welfare (THL); 2016.

3. Finlex. Tobacco Act 549/2016.

4. Finlex. Primary Health Care Act 66/1972.

5. Finlex. Local Government Act 410/2015.

6. Finlex. Health Care Act 1327/2010.

7. Finlex. Alcohol Act 1102/2017.

8. Finlex. Act on Supporting the Functional Capacity of the Older Population and on Social and Health Services for Older Persons 980/2012.

9. Finlex. Act on care services for the elderly 980/2012.

10. Borodulin K, Wennman H, Mäki-Opas T, Jousilahti P. The public health goals of WHO for increasing physical activity are achievable. Data Brief 23. Helsinki: National Institute for Health and Welfare (THL); 2015.

11. Karlsson T, Montonen M, Mäkelä P, Österberg E. The public health goals of WHO require consistent work to reduce alcohol-related harm. Data Brief 21/2015. Helsinki: National Institute for Health and Welfare (THL); 2015.

12. Vartiainen E, Laatikainen T, Jousilahti P, Tapanainen H. The target of WHO to lower the mortality rate is achievable - but the requisite is to lower the level of serum cholesterol. Data Brief 26/2015. Helsinki: National Institute for Health and Welfare (THL); 2015.

13. Peltonen M, Laatikainen T, Lindström J, Jousilahti P. WHO aims to stop the increase of obesity and type 2 diabetes action is needed in Finland. Data Brief 27/2015. Helsinki: National Institute for Health and Welfare (THL); 2015.

14. Kuulasmaa K, Tolonen H. WHO MONICA Project and its Connections to the North Karelia Project. Glob Heart 2016;11(2):217-221.

15. Laatikainen T, Jula A, Jousilahti P. The targets set by WHO to reduce blood pressure will not be reached without nutritional changes and more effective care. Data Brief 25/2015. Helsinki: National Institute for Health and Welfare (THL); 2015.

16. Heloma A, Ruokolainen O, Jousilahti P. WHO's goal to reduce smoking is achievable - the national Tobacco-Free Finland 2040 project requires stronger action. Data Brief 24/2015. Helsinki: National Institute for Health and Welfare (THL); 2015.

17. Melkas T. Health in all policies as a priority in Finnish health policy: a case study on national health policy development. Scand J Public Health 2013;41 Suppl 11:3-28.

18. Sainio P, Koskinen S, Sihvonen A, Martelin T, Aromaa A. Iäkkään väestön terveyden ja toimintakyvyn kehityslinjoja. In: Noro A, Alastalo H (eds.). Vanhuspalvelulain 980/2012 toimeenpanon seuranta. Tilanne ennen lain voimaantuloa vuonna 2013. Helsinki: THL; 2014. p. 37-41.

19. Ministry of Finance. Regional Government, Health and Social Services Reform: General information. Ministry of Finance, Finland. 2018. https://alueuudistus.fi/en/general-information-reform. Accessed 20 May 2018.

20. World Health Organization. Health 2020. A European policy framework and strategy for the 21st century. Copenhagen: The Regional Office for Europe of the World Health Organization; 2013.

21. Duodecim, Finland. Current Care Guidelines. 2018. http://www.kaypahoito.fi/web/kh/suositukset. Accessed 27 December 2018.

22. Terveyden ja Hyvinvoinnin laitos, Valtion Ravitsemusneuvottelukunta. Syödään yhdessä - ruokasuositukset lapsiperheille. Kide 26. Tampere: Juvenes Print – Suomen Yliopistopaino Oy; 2016.

23. Valtion Ravitsemusneuvottelukunta. Terveyttä ruoasta. Suomalaiset ravitsemussuositukset 2014. Helsinki: Valtion Ravitsemusneuvottelukunta; 2014.

24. Valsta LM, Tapanainen H, Sundvall J, et al. Explaining the 25-year decline of serum cholesterol by dietary changes and use of lipid-lowering medication in Finland. Public Health Nutr 2010;13(6A):932-938.

25. Opetusministeriö ja Nuori Suomi ry. Fyysisen aktiivisuuden suositus kouluikäisille 7-18 -vuotiaille. Recommendations for the physical activity of school-aged children. Helsinki: Nuori Suomi; 2008.

26. Opetus- ja kulttuuriministeriö. Iloa, leikkiä ja yhdessä tekemistä. Varhaisvuosien fyysisen aktiivisuuden suositukset. Opetus- ja kulttuuriministeriön julkaisuja 2016:21. Helsinki: Opetusministeriö; 2016.

27. Sosiaali- ja terveysministeriö. Ikääntyneiden ihmisten ohjatun terveysliikunnan laatusuositukset. Sosiaali- ja terveysministeriön oppaita 2004:6. Helsinki: Sosiaali- ja terveysministeriö; 2004.

28. Sosiaali- ja terveysministeriö, UKK-instituutti. Istu vähemmän - voi paremmin! Kansalliset suositukset istumisen vähentämiseen. Sosiaali- ja terveysministeriön esitteitä 2015. Helsinki: Edita Prima; 2015.

29. Sosiaali- ja terveysministeriö. Muutosta liikkeellä! Valtakunnalliset yhteiset linjaukset terveyttä ja hyvinvointia edistävään liikuntaan 2020. Sosiaali- ja terveysministeriön julkaisuja 2013:10. Tampere: Juvenes Print – Suomen Yliopistopaino Oy; 2013.

30. Sosiaali- ja terveysministeriö, Opetusministeriö, Liikenne- ja viestintäministeriö, Suomen Kuntaliitto. Suositukset liikunnan edistämiseksi kunnissa. Sosiaali- ja terveysministeriön esitteitä 2010:3. Helsinki: Yliopistopaino; 2010.

31. Karvinen E, Kalmari P, Koivumäki K. Ikäihmisten liikunnan kansallinen toimenpideohjelma. Liikunnasta terveyttä ja hyvinvointia. Opetus- ja kulttuuriministeriön julkaisuja 2011:30. Jyväskylä: Kopihyvä; 2011.

32. Suomen tuki –ja liikuntaelinliitto – Suomen Tule ry. Kansallinen TULE - ohjelma. Helsinki: Rakennuspaino Oy; 2007.

33. Terveyden ja Hyvinvoinnin laitos, (THL). Lihavuus laskuun - Hyvinvointia ravinnosta ja liikunnasta - Kansallinen lihavuusohjelma 2012-2015. Helsinki: Terveyden ja Hyvinvoinnin laitos; 2013.

34. Terveyden ja Hyvinvoinnin laitos, (THL). Lihavuus laskuun - Hyvinvointia ravinnosta ja liikunnasta: Kansallinen lihavuusohjelma - Toiminta- ja toimeenpanosuunnitelma 2016-2018. Työpaperi 18/2016. Helsinki: Terveyden ja hyvinvoinnin laitos; 2016.

35. Finnish Diabetes Association. Diabeteksen ehkäisyn ja hoidon kehittämisohjelma DEHKO 2000–2010. Loppuraportti. Pori: Kehitys Oy; 2011.

36. Finnish Diabetes Association. Programme for the Prevention of Type 2 Diabetes in Finland 2003-2010. Jyväskylä: Gummerus Printing; 2003.

37. Haahtela T, Valovirta E, Hannuksela M, von Hertzen L, Jantunen J, Kauppi P, Ketola T, Laatikainen T, Lindström I, Mäkinen-Kiljunen S, Linna M, Pajunen S, Pelkonen A, Petman L, Puolanne M, Repo I, Saarinen K, Savolainen J, Tommila E, Vasankari T, Mäkelä MJ. Kansallinen allergiaohjelma 2008–2018 puolivälissä – suunnanmuutos tuo tuloksia. Suomen Lääkärilehti 2015;35:2172d.

38. Sosiaali- ja terveysministeriö. Kansallinen muistiohjelma 2012–2020. Tavoitteena muistiystävällinen Suomi. Sosiaali- ja terveysministeriön raportteja ja muistioita 2012:10. Helsinki: Sosiaali- ja terveysministeriö; 2012.

39. Klemetti R, Raussi-Lehto E (eds.). Edistä, ehkäise, vaikuta - Seksuaali- ja lisääntymisterveyden toimintaohjelma 2014-2020. Tampere: Juvenes Print – Suomen Yliopistopaino Oy; 2014.

40. Sosiaali- ja terveysministeriö. Ehkäisevän päihdetyön toimintaohjelma. Alkoholi-, huume- ja rahapelihaittojen sekä tupakoinnin vähentäminen. Sosiaali- ja terveysministeriön julkaisuja 2015:19. Helsinki: Lönnberg Painot Oy; 2015.

41. Puska P, Vartiainen E, Nissinen A, Laatikainen T, Jousilahti P. Background, Principles, Implementation, and General Experiences of the North Karelia Project. Glob Heart 2016;11(2):173-178.

42. Vartiainen E, Puska P, Pekkanen J, Tuomilehto J, Jousilahti P. Changes in risk factors explain changes in mortality from ischaemic heart disease in Finland. BMJ 1994;309(6946):23-27.

43. Vartiainen E, Laatikainen T, Tapanainen H, Puska P. Changes in Serum Cholesterol and Diet in North Karelia and All Finland. Glob Heart 2016;11(2):179-184.

44. Jousilahti P, Laatikainen T, Salomaa V, Pietilä A, Vartiainen E, Puska P. 40-Year CHD Mortality Trends and the Role of Risk Factors in Mortality Decline: The North Karelia Project Experience. Glob Heart 2016;11(2):207-212.

45. Vartiainen E, Laatikainen T, Peltonen M, Puska P. Predicting Coronary Heart Disease and Stroke: The FINRISK Calculator. Glob Heart 2016;11(2):213-216.

46. University of Eastern Finland. Stopdia project. FINDRISK risk calculator for diabetes type 2: https://www.stopdia.fi/. 2018. Accessed 20 November 2018.

47. Lindström J, Tuomilehto J. The diabetes risk score: a practical tool to predict type 2 diabetes risk. Diabetes care 2003;26(3):725-731.

48. Kivipelto M, Ngandu T. From Heart Health to Brain Health: Legacy of the North Karelia Project for Dementia Research. Glob Heart 2016;11(2):235-242.

49. Schroderus T. Virallisia ravitsemussuosituksia kritisoiva Tomi Kokko kohauttaa jälleen - ”Kierrän kaikki Suomen kaupungit ja annan ihmisille ohjeet ruokaremonttiin”. Iltalehti 2018 February 21.

50. Jaaskelainen T, Itkonen ST, Lundqvist A, et al. The positive impact of general vitamin D food fortification policy on vitamin D status in a representative adult Finnish population: evidence from an 11-y follow-up based on standardized 25-hydroxyvitamin D data. Am J Clin Nutr 2017;105(6):1512-1520.

51. Valio, Finland. Lactose-free products: https://www.valio.com/consumers/lactose-free/. 2018. Accessed 2 January 2019.

52. Gylling H, Hallikainen M, Nissinen MJ, Miettinen TA. The effect of a very high daily plant stanol ester intake on serum lipids, carotenoids, and fat-soluble vitamins. Clin Nutr 2010;29(1):112-118.

53. Castro Cabezas M, de Vries JH, Van Oostrom AJ, Iestra J, van Staveren WA. Effects of a stanol-enriched diet on plasma cholesterol and triglycerides in patients treated with statins. J Am Diet Assoc 2006;106(10):1564-1569.

54. The Finnish Heart Association, Finland. Sydänmerkki - Heart Symbol: http://www.sydanmerkki.fi/en/. 2018. Accessed 1 December 2018.

55. Lahti-Koski M, Helakorpi S, Olli M, Vartiainen E, Puska P. Awareness and use of the Heart Symbol by Finnish consumers. Public Health Nutr 2012 March 01;15(3):476-482.

56. Puska P, Laatikainen T, Korpelainen V, Vartiainen E. Contribution of the North Karelia Project to International Work in CVD and NCD Prevention and Health Promotion. Glob Heart 2016;11(2):243-246.

57. Kilpeläinen K, Aromaa A, ECHIM Core Croup (eds.). European health indicators: development and initial implementation: final report of the ECHIM project. Publications of National Public Health Institute B 31 /2008. Helsinki: Helsinki University Press; 2008.

58. Tuomi-Nikula A, Gissler M, Sihvonen A-P, Kilpeläinen K, ECHIM Core Group (eds.). Implementation of European Health Indicators - First years. Publications of National Public Health Institute 49/2012. Tampere: Juvenes Print - Tampere University Print; 2012.

59. Tolonen H, Koponen P, Al-Kerwi A, Capkova N, Giampaoli S, Mindell J, et al. European health examination surveys - a tool for collecting objective information about the health of the population. Arch Public Health 2018;76:4.

60. Tolonen H, Koponen P, Mindell J, Mannisto S, Kuulasmaa K. European Health Examination Survey -towards a sustainable monitoring system. Eur J Public Health 2014;24(2):338-344.

61. Tunstall-Pedoe H, World Health Organization MONICA Project. MONICA, monograph, and multimedia sourcebook: world's largest study of heart disease, stroke, risk factors, and population trends 1979-2002. Geneva: World Health Organization; 2003.

62. Morgenstern W. CINDI, Countrywide Integrated Noncommunicable Diseases Intervention Programme: baseline evaluation. Berlin: New York: Springer-Verlag; 1991.

63. Prättälä R, Helakorpi S, Sipilä N, Sippola R, Sääksjärvi K. Social determinants of health behaviours: Finbalt Health Monitor 1998-2008. Report of National Institute for Health and Welfare 25/2011. Tampere: Juvenes Print; 2011.

64. The National Institute for Health and Welfare, Finland. Welfare compass. 2018. https://www.hyvinvointikompassi.fi/en/web/hyvinvointikompassi/. 2018. Accessed 18 November 2018.

65. The National Institute for Health and Welfare, Finland. Terveytemme.fi. 2018. http://www.terveytemme.fi/. Accessed 19 November 2018.

66. The National Institute for Health and Welfare, Finland. Sotkanet.fi. 2018. https://sotkanet.fi/sotkanet/en/index?. Accessed 19 November 2018.

67. Lundqvist A, Mäki-Opas T (eds.). Health 2011 Survey - Methods. National Institute for Health and Welfare. Report 8/2016. Tampere: Juvenes Print – Finnish University Print Ltd; 2016.

68. Murto J, Pentala O, Koskela T, Jussmäki T. Poimintoja aikuisväestön terveydestä, hyvinvoinnista ja elinoloista Suomessa 2013-2016 - ATH-tutkimuksen tuloksia. Data brief 7/2017. Helsinki: National Institute for Health and Welfare (THL); 2017.

69. Konttinen H, Silventoinen K, Sarlio-Lahteenkorva S, Mannisto S, Haukkala A. Emotional eating and physical activity self-efficacy as pathways in the association between depressive symptoms and adiposity indicators. Am J Clin Nutr 2010;92(5):1031-1039.

70. Turunen A et al. Fish in Diet: Results from the Fishermen study and the Health 2000 survey. National Institute for Health and Welfare. Report 25/2009. Helsinki: Yliopistopaino Oy; 2009.

71. Turunen A. Epidemiological studies on fish consumption and cardiovascular health - Results from the Fishermen study and the Health 2000 survey. National Institute for Health and Welfare. Research 79/2012. Tampere: Juvenes Print - University Press.

72. Jousilahti P, Laatikainen T, Haahtela T, Vartiainen E. Astma ja hengitystieallergiat ovat lisääntyneet Suomessa - allergiaohjelma pyrkii taittamaan kasvun. Data brief 5/2016. Helsinki: National Institute for Health and Welfare (THL); 2017.

73. Skogberg N, Laatikainen T, Koskinen S, Vartiainen E, Jula A, Leiviska J, et al. Cardiovascular risk factors among Russian, Somali and Kurdish migrants in comparison with the general Finnish population. Eur J Public Health 2016;26(4):667-673.

74. Castaneda AE, Rask S, Koponen P, Mölsä M, Koskinen S. Maahanmuuttajien terveys ja hyvinvointi - Tutkimus venäläis-, somalialais- ja kurditaustaisista Suomessa. Report of National

Institute for Health and Welfare 61/2012. Helsinki: National Institute for Health and Welfare (THL); 2012.

75. Rask S, Suvisaari J, Koskinen S, Koponen P, Molsa M, Lehtisalo R, et al. The ethnic gap in mental health: A population-based study of Russian, Somali and Kurdish origin migrants in Finland. Scand J Public Health 2016;44(3):281-290.

76 Abbasi A. et al. Prediction models for risk of developing type 2 diabetes: systematic literature search and independent external validation study. BMJ 2012;345:e5900.

77. Salomaa V. Genetic and Environmental Contributions to Cardiovascular Risk: Lessons from North Karelia and FINRISK. Glob Heart 2016;11(2):229-233.

78. Repas TB, Tanner JR. Preventing early cardiovascular death in patients with familial hypercholesterolemia. J Am Osteopath Assoc 2014;114(2):99-108.

79. Lahtinen AM, Havulinna AS, Jula A, Salomaa V, Kontula K. Prevalence and clinical correlates of familial hypercholesterolemia founder mutations in the general population. Atherosclerosis 2015;238(1):64-69.

(80) Norio R. Suomi-neidon geenit. Helsinki: Otava; 2000.

81. Vos T, et al. Global, regional, and national incidence, prevalence, and years lived with disability for 328 diseases and injuries for 195 countries, 1990–2016: a systematic analysis for the Global Burden of Disease Study 2016. Lancet 2017;390(10100):1211-59.

82. Borodulin K, Tolonen H, Jousilahti P, et al. Cohort Profile: The National FINRISK Study. Int J Epidemiol 2018;47(3): 696-696i.

83. National Institute for Health and Welfare, Finland. Finnish food composition database Fineli. 2018. https://fineli.fi/fineli/en/index?. Accessed 19 October 2018.

84. Rategia, Finland. Miils-application: https://www.miils.com/accounts/login?next=/home. 2018. Accessed 3 November 2018.
